# Development and Validation of Waist Girth-Based Equations to Evaluate Body Composition in Colombian Adults: Rationale and STROBE–Nut-Based Protocol of the F20 Project

**DOI:** 10.3390/ijerph191710690

**Published:** 2022-08-27

**Authors:** Diego A. Bonilla, Leidy T. Duque-Zuluaga, Laura P. Muñoz-Urrego, Yurany Moreno, Jorge M. Vélez-Gutiérrez, Katherine Franco-Hoyos, Alejandra Agudelo-Martínez, Gustavo Humeres, Richard B. Kreider, Jorge L. Petro

**Affiliations:** 1 Research Division, Dynamical Business & Science Society—DBSS International SAS, Bogota 110311, Colombia; 2 Grupo de Investigación NUTRAL, Facultad Ciencias de la Nutrición y los Alimentos, Universidad CES, Medellin 050021, Colombia; 3 Research Group in Physical Activity, Sports and Health Sciences (GICAFS), Universidad de Córdoba, Monteria 230002, Colombia; 4 Sport Genomics Research Group, Department of Genetics, Physical Anthropology and Animal Physiology, Faculty of Science and Technology, University of the Basque Country (UPV/EHU), 48940 Leioa, Spain; 5 ARTHROS Centro de Fisioterapia y Ejercicio, Medellin 050012, Colombia; 6 Instituto de Ciencias de Rehabilitación y el Movimiento (ICRM), Universidad Nacional de San Martín, Buenos Aires 1650, Argentina; 7 Exercise & Sport Nutrition Laboratory, Human Clinical Research Facility, Texas A&M University, College Station, TX 77843, USA

**Keywords:** waist circumference, regression equations, fat mass, fat free mass, kinanthropometry, health, disease, sports nutrition, exercise performance

## Abstract

Waist girth (WG) represents a quick, simple, and inexpensive tool that correlates with excess of fat mass in humans; however, this measurement does not provide information on body composition. The evaluation of body composition is one of the main components in the assessment of nutritional status. Indeed, the use of anthropometry-based equations to estimate body fat and fat-free mass is a frequent strategy. Considering the lack of validation in the Colombian population, the aim of this research study (the F20 Project) is to externally validate WG-based equations (e.g., relative fat mass), and also to develop and validate new models that include WG to estimate body composition in Colombian adults compared to DXA. This cross-sectional study will be carried out following the guidelines for Strengthening the Reporting of Observational Studies in Epidemiology–Nutritional Epidemiology (STROBE–nut). Using stratified probabilistic sampling, the study population will be adults with different levels of physical activity residing in Medellín and its metropolitan area. The results of this study will not only validate the estimation performance of the current WG-based equations, but they will also develop new equations to estimate body composition in the Colombian population. This will improve professional practice in health, exercise, and sports sciences (ClinicalTrials.gov ID #NCT05450588).

## 1. Introduction

The evaluation of body composition is one of the main components in the assessment of nutritional status under the ABCDE model, which considers anthropometric, biochemical, clinical, dietary, and environmental (i.e., socioeconomic status, lifestyle, etc.) aspects as fundamental when analyzing an individual. In fact, considering that the nutritional status of an individual is influenced by endogenous and exogenous factors, the ABCDE model is recommended and valid in both community and clinical nutritional practice [1], as well as at the sports level [2]. Although obesity is currently defined as a “chronic, progressive, relapsing, and treatable multi-factorial, neurobehavioral disease, wherein an increase in body fat promotes adipose tissue dysfunction and abnormal fat mass physical forces, resulting in adverse metabolic, biomechanical, and psychosocial health consequences” [3], the criteria currently used do not allow for the identification of excess adipose tissue. The Body Mass Index (BMI) is one of the most widely used anthropometric indices for predicting nutritional status and the classification of obesity; however, it presents certain errors in its interpretation since it does not differentiate the body composition of a subject, but only relates body mass and stature [4,5]. The ratio of body mass to stature squared has been shown to be useful for large populations; however, adiposity only increases as a function of stature in children, but in adults it is influenced by lean mass. Therefore, it is currently claimed that BMI is not a valid indicator of an individual’s nutritional status [6].

Various assessment methods are used to evaluate body composition, including direct (dissection of cadavers), indirect (magnetic resonance imaging (MRI); computerized axial tomography (CAT); dual-energy X-ray absorptiometry (DXA)) and doubly indirect methods (anthropometry and bioelectrical impedance analysis (BIA)) [7]. Currently, the four-component model (4C Model) involves an independent assessment of body density, total body water, and bone-mineral content, and is therefore commonly described as the most accurate measure because it combines the advantages of different methods [8]. Although there are several indirect methods that allow a more accurate evaluation of body composition and density, in practice there are certain limitations related to the high cost of the equipment, the control of radiation exposure, and the technical maintenance/specialization required for its use [9]. In this regard, anthropometric measurements (skinfolds, girths, lengths, and diameters) have been used to evaluate morphology, body composition, and maturation status, among other aspects. Therefore, anthropometry provides practitioners with a tool that serves to monitor physical performance or health-related outcomes, while taking into account the particular characteristics of the individuals.

### 1.1. Background Rationale

Kinanthropometry is a discipline that analyzes the use of body measurements and their relationships with other parameters of health or human movement. It is considered to be a simple technique that also helps to estimate body composition (e.g., body fat percentage) from regression equations [10]. Among the anthropometric variables that have been used for the development of the most common equations in current daily practice (i.e., body mass, stature, age, sex, and skinfolds), it should be noted that waist girth (WG) has been used to a lesser extent, even though it represents a quick, simple, and inexpensive tool for predicting excess fat mass in humans [11]. The WG is the girth at the narrowest position in the abdominal area between the tenth rib and the iliac crest, as described in the International Standards for Anthropometric Assessment established by the International Society for the Advancement of Kinanthropometry (ISAK) [12]. WG is considered a determining variable in the diagnosis of metabolic syndrome as it is directly related to visceral fat [13] and has stronger associations with health-risk indicators [14]. WG has been used to diagnose abdominal obesity and as an indicator of cardiovascular risk [15,16,17]. Although this measurement cannot provide information on body composition, absolute values of this girth have shown a high correlation with the percentage of body fat obtained by DXA, which has positioned WG as an optimal indicator for the evaluation of body fat in different populations [18,19,20,21]. The presence of central obesity is defined based on the proposed cutoff for Latin-American adults (WG ≥ 90 cm in women and WG ≥ 94 cm in men) [22].

Several authors have concluded that regression equations that use WG as a predictive variable appear to estimate body fat percentage more accurately [23,24,25]. In this sense, the relatively recent efforts of some research groups have developed equations that include WG as an independent variable to estimate fat mass and fat-free mass. On the one hand, Woolcott and Bergman [26] developed and validated a simple equation, named relative fat mass (RFM), to estimate the percentage of total body fat using the WG, stature, and sex of the American adult population (64 − (20 × (stature [m]/WG [m])) +  (12 × sex [0 for male and 1 for female])). The population data were extracted from the National Health and Nutrition Examination Survey (NHANES) between 1999 and 2006. The performance of RFM as an easy and useful tool in clinical practice, with a better correlation to DXA than BMI (RFM: R^2^ = 0.84; RMSE = 3.51% vs. BMI: R^2^ = 0.36; RMSE = 7.04%) has been validated in adults [27], children between 8 and 14 years old, and adolescents between 15 and 19 years old [28], obese subjects [29,30], and individuals with Down syndrome [31], among other populations at cardiovascular risk [32,33]. It is worth noting that the RFM defines obesity according to the cutoff points of the DXA measurements, to the point that following the rounded thresholds of the RFM baseline, the current recommendation for diagnosing obesity and predicting the risk of death with this parameter is 40% for women and 30% for men [34]. On the other hand, Takai et al. [35] developed an equation to estimate fat-free mass in male athletes using some indices as independent variables, including WG/body mass and stature/WG. The equation developed by these authors was validated with a sample of 48 athletes from different disciplines and showed a high correlation (R^2^ = 0.900, SEE = 2.3 kg [3.8%]) with the fat-free-mass data obtained by DXA (FFM (kg) = 0.883 × Body mass/WG [kg/m] + 43.674 × WG/Stature [cm/cm] − 41.480). The results of this study demonstrated that an equation using body mass and stature ratios with WG as independent variables is applicable and useful for predicting fat-free mass in male athletes.

In Colombia, there are no studies that have validated these WG-based equations for estimating body composition. A recent study performed the concurrent validation of 5 equations frequently used to estimate the percentage of body fat in young Colombian athletes of both sexes (Slaughter, Durnin–Rahaman, Lohman, Johnston, and the 5C Model by Kerr) when comparing the results against DXA measurement [36]. Interestingly, the Durnin–Rahaman and Johnston equations showed good-to-excellent validity with low bias in the population analyzed, but none of these equations used WG as a predictor variable. Only one study in Colombian adult women utilized anthropometric data (body mass, stature, skinfolds, and girths (including WG)) and hydrodensitometry evaluation to subsequently develop different linear regression models to estimate the percentage of body fat [37]. Cross-validation of the developed models showed that both Equation (1) (SEE = 2.84%; R^2^ = 0.71) and Equation (2) (SEE = 3.06%; R^2^ = 0.67) had a low standard of error in the estimation of the percentage of body fat and moderate–high values in the coefficient of determination. However, this research has two important limitations that are worth mentioning in regard to generalizability and interpretation. First, the research used Lohman’s anthropometric protocol—instead of the current ISAK recommendations that are promoted by international entities, such as the World Health Organization [38] and the International Olympic Committee [39]; in fact, this protocol is less reproducible/standardized and less recommended, and it is increasingly falling out of use because it includes invasive measurements, such as the midaxillary skinfold—which was a variable in one of the generated models. Second, hydrodensitometry as a reference methodology has some disadvantages, including the exclusion of participants who do not complete the underwater weighing test, as well as the fact that it is a methodology for estimating body density but not for estimating fat percentage. Importantly, the use of the Siri equation to estimate body-fat percentage implicates an additional estimation, so the values reported may not be accurate [40].

### 1.2. Objectives

The use of equations to estimate body composition is a frequent strategy since it represents a doubly indirect method with a good correlation to the methods mentioned above. However, this leads to the question of whether current WG-based equations are valid for estimating fat mass and fat-free mass in the Colombian population with different levels of physical activity? On the other hand, would it be advantageous to develop new models to obtain a specific equation for the Colombian population? Thus, the aim of this study will be twofold: (i) externally validate the current WG-based equations to estimate fat mass and fat-free mass as compared with DXA, and (ii) develop new models to estimate fat mass and fat-free mass using the WG as a predictive variable, and subsequently validate the new equations in the Colombian population with different levels of physical activity.

## 2. Methods

### 2.1. Study Design

This will be a cross-sectional study based on the Strengthening the Reporting of Observational Studies in Epidemiology–Nutritional Epidemiology (STROBE–nut) guidelines, an Extension of the STROBE Statement [41]. This study (the F20 Project) will test the validity of current WG-based equations and will develop new models to estimate fat mass and fat-free mass as compared with DXA as a reference criterion.

### 2.2. Setting

Anthropometric data will be collected from the participants who fulfill the selection criteria between the first and second semesters of the 2023 academic year. This research will be conducted with the support of the Universidad CES as part of the thesis activities required by the Master of Science in Sports Nutrition degree program. Experimental procedures for developing and cross-validating the new equations will be conducted, as in previous studies carried out by our research group [42,43].

### 2.3. Participants

The study population will be healthy, male and female residents of Medellín and the nearby municipalities that make up its metropolitan area (the Andean Region of Colombia), who exhibit different levels of physical activity. The sample of the sedentary population will be obtained through an open call to both students and administrative personnel at Universidad CES. The sample of the physically active population will be obtained from the collaboration agreement between DBSS International and Smart Fit Colombia, for which a sample of personalized trainers working in Medellín and its metropolitan area will be used. Finally, the sample of professional athletes will be obtained by taking advantage of the interinstitutional relations of the Research Division of DBSS International and the IPS ARTHROS—Physiotherapy and Exercise Center with clubs and sports entities in Antioquia.

In order to comply with the objectives of this research, the following will be taken into consideration as inclusion criteria: (i) +18 years old (under 60 years); (ii) residing in the city of Medellín or nearby municipalities of the metropolitan area; (iii) low, moderate, and high categories, according to the IPAQ-SF questionnaire [44] for sedentary, physically active, and athletes, respectively. In addition, athletes will need to demonstrate previous experience in sports events (>1 year) and current affiliation with a club team participating in league games or sanctioned tournaments; (iv) signed, informed consent to submit to the anthropometric and DXA measurements. The following participants will be excluded: (i) those individuals diagnosed with a disease and/or a special condition that limits exposure to radiation emitted by DXA; (ii) adults over 60 years of age; (iii) pregnant women; (iv) athletes with injuries; and (v) people with implants or prostheses. All procedures will be developed in accordance with the latest version of the Declaration of Helsinki [45]. The study protocol has been approved by the Research and Innovation Committee at the University CES (Acta0031Proy115TG) and registered with ClinicalTrials.gov (ID #NCT05450588).

### 2.4. Variables

As reference criteria, whole and regional body composition will be estimated using DXA. The restricted-profile anthropometric variables established by ISAK will be measured: basic measurements; skinfolds (mm); girths (cm); lengths and heights (cm); and breadths and depths (cm). We will also estimate body composition based on anthropometric data.

### 2.5. Data Sources/Measurement

All of the measurements for the selected participants will be performed at the research center CESNUTRAL at CES University and the facilities of the IPS ARTHROS—Physiotherapy and Exercise Center (Medellín, Colombia). To reduce technical errors in measurement, the evaluations will be conducted between 9:00 and 17:00 (GMT-5) at controlled environmental conditions (<24 °C and <60% humidity).

#### 2.5.1. Body Composition

DXA measurements will be performed following all current recommendations and the laboratory procedures reported in previous articles published by our research group [46,47,48]. To perform the scans, a Lunar Prodigy™ unit will be used (General Electric Healthcare, Madison, WI, USA). Each subject will be scanned by a certified bone densitometry technologist, and the distinguished bone and soft tissue, edge detection, and regional demarcations will be performed using computer algorithms.

#### 2.5.2. Anthropometry

All anthropometric measurements will be carried out in accordance with the International Standards for Anthropometric Assessment published by the ISAK [12]. Body mass will be measured with a digital scale to the nearest 100 g (Seca 874, Hamburg, Germany). A portable stadiometer with a 1 mm graduation will be used to measure stature and wingspan (Seca 213, Hamburg, Germany). A 50 cm high wooden anthropometric box will be used to measure the sitting height. The skinfold thicknesses will be measured with a calibrated skinfold caliper (Harpenden, UK). Girths will be measured with a non-extensible metal tape with a thickness of 0.7 cm (Lufkin w606PM, Apex Tool Group, Sparks, MD, USA). The measurement of breadths will be performed with a Realmet Petit (16 cm) small sliding caliper (Realmet, Barcelona, Spain). Averages based on two measurements of anthropometric data will be calculated and analyzed.

#### 2.5.3. Anthropometry-Based Analysis of Body Composition

We will estimate whole-body-fat percentage (as RFM) in sedentary and physically active individuals following procedures conducted by Woolcott and Bergman [26]. The Takai et al., equation [35] will be used to estimate fat-free mass in athletes. We will also report the sum of skinfolds (∑S) as an absolute variable (expressed in millimeters) which not only gives information about the local distribution of subcutaneous fat tissue, but which also indicates whole-body adiposity since it correlates with whole-body-fat mass [40,49]. As a musculoskeletal index, the skinfold-corrected muscle girths for arm, chest, waist, thigh, and calf will be calculated according to the expression: girth—(π × skinfold) [50].

### 2.6. Bias

The different types of intentional or unintentional bias and error that may occur in this study will be controlled according to their nature: (i) Gross errors—daily calibrated, certified, and recognized by international entities or Colombian technical regulations and legal metrology (Superintendence of Industry and Commerce) equipment and instruments will be used; (ii) Pre-analytical errors (blunders)—the research assistance personnel will be previously trained in the annotation and accompaniment at the moment of recording the data using a specific typography and computer files for databases. The recorder assistant will always repeat the value dictated by the anthropometrist at the time of the assessment; (iii) Systematic errors—the relative technical error of measurement of the technicians will be reported. We will ensure that the intra-observer technical error of measurement of the certified anthropometrists (√[∑differences^2^/2n]) will be less than 7.5% for skinfolds and less than 1.5% for the other measurements, which is considered acceptable by the ISAK recommendations [10]. The coefficient of variation of the DXA equipment will be reported (CV% = Standard Deviation/Average × 100%). The test–retest reliability of the DXA unit that will be used in this study has shown a coefficient of variation ranging from 1.0 to 2.0%. In addition, similar to previous reports [46], we will adjust the DXA measurements based on the model proposed by Heymsfield et al. [51] to eliminate the influence of fat-free adipose tissue (FFAT) on DXA-derived fat-free mass. This has been shown to provide more accurate values to detect changes in body composition [52]. Therefore, we will estimate the adipose tissue as DXA-fat mass ÷ 0.85; FFAT will be then calculated as adipose tissue × 0.15; and, finally, DXA-derived fat-free mass will be adjusted with the subtraction of FFAT.

### 2.7. Study Size

According to Knofczynski and Mundfrom [53], to obtain (at least) a coefficient of determination (R^2^) of 0.5 with an excellent level of prediction using 3 independent variables, a total of 130 participants will be needed. Stratified probability sampling techniques will be used. Thus, the total number of participants will be divided into three strata following optimal allocation methods. A fixed cost has been assigned for each stratum (sedentary persons, C1; physically active subjects, C2; and athletes, C3, given that C3 > C2 > C1) to calculate the sample size of each population using Lagrange multipliers, given by the expression:

(1) $$nh=n\;\frac{NhSh/\surd Ch}{\sum_{h=1}^{L}NhSh/\surd Ch},\;h=1,\;2,\;\ldots ,\;L$$

where, the values of *n_h_* correspond to the sample units for each stratum, *N_h_* is the maximum number of participants allowed, *C_h_* is the constant cost in all strata, *L* is the number of strata, and *S_h_* is the population quasi-variance of stratum *h*. The values obtained for the sedentary group are *n* = 54; for the physically active population group, *n* = 43; and for the athlete group, *n* = 33. To validate the new equation, the development/validation ratio will be 70/30 over the number of subjects participating in the study.

### 2.8. Statistical Methods

The descriptive analysis of the anthropometric characteristics of the study population will be reported as means, standard deviations, and 95% confidence intervals. First, the total sample of participants will be used for the external validation of the RFM (to estimate whole-body-fat percentage) [26] and the Takai et al., equation (to estimate fat-free mass) [35]. A correlation analysis will be performed by calculating Pearson’s r, intraclass correlation coefficient (ICC), concordance correlation coefficient (ρ_c_), adjusted coefficient of determination (aR^2^), and root mean squared error (RMSE) as compared to DXA measurements. Smaller values of standard error of the estimate (SEE) and RMSE will indicate that the estimated values are closer to the DXA measurements. Bland–Altman diagrams will be used for the concordance analysis. This is a procedure to determine whether two measurement methods, X and Y, agree sufficiently to be declared interchangeable (D = X − Y). The mean of these differences represents the systematic error (bias), while the variance of these differences (1.96 SD) measures the dispersion of the random error.

The development of the new equations to estimate fat mass (dependent variable) from different combinations of WG with sex, physical activity level, body mass, and stature (as predictor variables) will be carried out with multiple regression models under traditional (Ordinary Least Squares method) and Bayesian approaches (limited to three independent variables). For the latter, the ‘bayesbr’ and ‘bayes.lm’ R packages will be used. Variance inflation factors will be used to detect multicollinearity among predictors in the multiple linear regression models, and autocorrelation in the residuals will be assessed through the Durbin–Watson test. The variation explained by the model will be determined by the aR^2^. Finally, the SEE will be calculated for all generated models to measure the regression’s precision, while the RMSE will be used to evaluate how close the estimated values from each equation are to the actual measured values as reported elsewhere [42]. All possible regression models will be ranked using the akaike information criterion (AIC), the Bayesian information criterion (BIC), the Mallows’ C_p_, and the Hocking’s Sp. After compliance with all of the assumptions of the multiple regression analysis (normality of residual errors will be confirmed with the Omnibus k-squared and Jarque-Bera tests), the model with the better performance that includes WG will be selected for further analysis. The predictability of the equation in the selected model will be tested in the validation sample by calculating the aR^2^, RMSE, CCC, and ICC with its respective 95% CI. The concordance analysis will be performed by means of Bland–Altman diagrams, reporting the concordance intervals at 95%. Statistical tests will be carried out using the SPSS v26 statistical package (IBM Corp., Armonk, NY, USA) and the latest version of the environment for statistical computing R [54].

## 3. Expected Results

Diagnosing nutritional status generally involves the use of two indicators—body mass and stature—which, although necessary, cannot define body composition accurately. Currently, WG is used as a better predictor of cardiovascular risk and central obesity. Interestingly, scientific evidence supports the predictive potential of WG given the high correlation of WG-based equations with more reference methods (e.g., DXA and CT) for the assessment of body composition. However, these also have limitations that are mainly due to differences with respect to the population on which they have been developed since they are specific and, given the fluctuations that occur in age and sex, further external validation is warranted [40,55]. Development and validation of new models will increase generalizability and scientific soundness at the time of evaluating body composition in the Colombian population with different levels of physical activity. Thus, future research like this should take advantage of the predictive potential of WG together with other basic measurements (body mass and stature) to estimate with a low rate of error the fat mass and fat-free mass in different populations. Importantly, evaluating the performance of new models with different combinations of several variables (e.g., age, sex, body mass, stature, and WG) raises its relevance. Based on the available literature, we expect to find, after model specification, the body-mass-to-WG and WG-to-stature ratios as part of the best regression model. The body-mass-to-WG ratio has been previously found to have a strong correlation with body composition across the lifespan [56,57]. On the other hand, the WG-to-stature ratio has been reported as a better predictor of abdominal-fat distribution in men compared to BMI or the WG-to-hip ratio using computed tomography as a standard method [58]. In fact, some recent research suggests the WG-to-stature ratio is a strong predictor of abdominal obesity with no relevant age- or sex-dependent effects [59,60,61,62].

## 4. Conclusions

The importance of this study protocol (the F20 Project) is the validation of both existing and new models that might result in fewer estimation errors regarding body composition. This will contribute to the generation of public health alerts and strategies for promotion and prevention since the results obtained will provide more accurate diagnoses of nutritional statuses as compared to those currently in use (e.g., BMI). Hence, the results of the present project will be of great help in evaluating the nutritional status, at the individual and collective levels, of different populations in Colombia. Aware of the need for external validation, we expect to obtain an accessible and easy-to-apply tool in clinical nutrition, sports, and public health for the estimation of fat mass and fat-free mass. We consider that the simple, validated/developed equations might become formally established tools for use by the Colombian health system.

## Data Availability

Not applicable.
